# Functional Connectivity in Islets of Langerhans from Mouse Pancreas Tissue Slices

**DOI:** 10.1371/journal.pcbi.1002923

**Published:** 2013-02-28

**Authors:** Andraž Stožer, Marko Gosak, Jurij Dolenšek, Matjaž Perc, Marko Marhl, Marjan Slak Rupnik, Dean Korošak

**Affiliations:** 1 Institute of Physiology, Faculty of Medicine, University of Maribor, Maribor, Slovenia; 2 Department of Physics, Faculty of Natural Sciences and Mathematics, University of Maribor, Maribor, Slovenia; 3 Faculty of Civil Engineering, University of Maribor, Maribor, Slovenia; 4 Faculty of Education, University of Maribor, Maribor, Slovenia; 5 CIPKeBiP-Centre of Excellence for Integrated Approaches in Chemistry and Biology of Proteins, Ljubljana, Slovenia; 6 CAMTP - Center for Applied Mathematics and Theoretical Physics, University of Maribor, Maribor, Slovenia; Princeton University, United States of America

## Abstract

We propose a network representation of electrically coupled beta cells in islets of Langerhans. Beta cells are functionally connected on the basis of correlations between calcium dynamics of individual cells, obtained by means of confocal laser-scanning calcium imaging in islets from acute mouse pancreas tissue slices. Obtained functional networks are analyzed in the light of known structural and physiological properties of islets. Focusing on the temporal evolution of the network under stimulation with glucose, we show that the dynamics are more correlated under stimulation than under non-stimulated conditions and that the highest overall correlation, largely independent of Euclidean distances between cells, is observed in the activation and deactivation phases when cells are driven by the external stimulus. Moreover, we find that the range of interactions in networks during activity shows a clear dependence on the Euclidean distance, lending support to previous observations that beta cells are synchronized via calcium waves spreading throughout islets. Most interestingly, the functional connectivity patterns between beta cells exhibit small-world properties, suggesting that beta cells do not form a homogeneous geometric network but are connected in a functionally more efficient way. Presented results provide support for the existing knowledge of beta cell physiology from a network perspective and shed important new light on the functional organization of beta cell syncitia whose structural topology is probably not as trivial as believed so far.

## Author Summary

Complex network theory has provided new tools for studying the structure and function of complex systems. A particularly attractive avenue in this context is the analysis of biological systems, since structural principles of complex networks have been identified at all scales of functioning of living organisms. In the present paper, we propose a construction of a complex network representation of pancreatic islets of Langerhans. In this microorgan, interconnected beta cells produce and secrete insulin, an anabolic hormone that controls the level of nutrients in the blood. We determine the functional connectivity on the basis of patterns of correlations between experimentally measured calcium dynamics in individual beta cells. The extracted pattern of pairwise interactions between network elements, i.e. beta cells, is then scrutinized with conventional tools for network analysis. Our findings are largely reconcilable with known structural and functional properties but also point to the presence of unexpected small-world attributes that appear to represent a general organizational principle of the functional connectivity between beta cells. We argue that complex network analysis applied to islets of Langerhans is a valuable new tool in the physiologist's analytical repertoire, and in the future it could help deepen our understanding of their physiology.

## Introduction

Over the last decade, a new field of network science has emerged and distinguished itself from preceding work in the realm of graph theory by focusing on real-world networks and by understanding networks as structures that can evolve in time and as frameworks upon which dynamical systems can be distributed [1], [2]. Most importantly, it has recognized the importance of building on both empirical observation and modeling for the development of new graph-theoretic models and for the understanding of experimental findings [2], [3]. Due to its deep experimental roots and a powerful armamentarium of analytical tools, network theory has become integral in the study of complex systems.

Employing it, we are beginning to understand structural properties and functional behaviors of systems at scales inaccessible to more classical approaches that handle complexity by explaining structure and function of individual parts. Beyond that, we are discovering common properties of real-world systems as diverse as biological, computer, technical, communication, and social networks [4]–[6]. The most abundantly present and functionally important properties in these networks are the so called small-world-ness [7] and scale-freeness [8]. If a network satisfies the criteria required for either or both, it is endowed with some characteristic properties. Small worlds display short internodal distances and highly clustered organization. In scale-free networks, the degree distribution follows a power law, meaning that these systems have no typical or mean node degree, i.e. they are scale invariant. In real physically embedded networks, there are constraints limiting the constant addition of new links and preferential attachment to the most connected vertices, leading to a cutoff of the power law regime in the connectivity distribution or making it disappear altogether [9], [10]. As a result, the so called broad-scale or single-scale small-world networks emerge [9], [11].

The presence of a small-world topology implies particular dynamic properties, such as stability, local and global efficiency in the interaction of their vertices, e.g. high signal-propagation speed and a high degree of synchronizability [7], [12]. Such characteristics have many already established and possible advantages for a number of complex systems, in particular for living organisms. To date, studies taking a graph-theoretical approach to analyze biological systems have predominantly dealt with intracellular metabolic networks [13]–[15], protein-protein interactions [14], [16], [17], signaling networks [14], [18], [19], transcription-regulatory networks [14], [20], interconnectedness of human diseases [21], multi-target drug design [22], and neuronal networks, with emphasis on brain network organization [23]–[25]. Since network science essentially relies on empirical data, the above repertoire reveals not only areas of greatest practical importance, but is essentially limited to those fields of interest where high-throughput experimental procedures and large databases, such as microarrays, proteomic tools, electronic patient records, functional magnetic resonance, electroencephalography etc. are available. These offer a supply for the increasing demands of network science for experimental evidence and conformation of predicted properties, fuelling its progress in leaps and bounds.

However, with the exception of some of the neurophysiological endeavors, so far all of the above applications gave only snapshot-like insights into complex systems. Due to the very nature of experimental methods they intrinsically missed out a system's temporal evolution and the fact that the nodes *per se* can be dynamical systems. In the spirit of systems biology, these two aspects, however, are of critical importance when it comes to the understanding of the way living systems are organized and how their anatomy supports their function. Even in neuroscience which of all life-sciences has come furthest in graph-theoretical applications and has benefited most from employing network approaches to structural and functional data, the above issues have been dealt with only partially. Namely, at the lowest, cellular level of investigation, structural data were used, inherently lacking the temporal and dynamical components, whereas functional data, e.g. from functional magnetic resonance imaging, electroencephalography or electromagnetography, capture the behavior on a significantly higher level of organization. Additionally, the latter always describe the underlying system at the level of sensors, making determination of source intrinsically complicated [26]–[29].

Functional studies on other tissues and at the lowermost level, where individual cells are the nodes considered, are only beginning to emerge [30]. Endocrine tissues are often organized as networks, evolve in time and their cells can be regarded as dynamical systems. Also, endocrine tissues are easier to probe than neural networks, yet first studies of pituitary gland structural and functional connectivity were conducted only recently [30]–[33]. This can at least partially be ascribed to the lack of experimental approaches that would enable us to assess the function of a large number of cells simultaneously, as noninvasively as possible and over long periods of time.

Considering the advantages conferred by small-world properties and emerging evidence on their presence in real biological systems, our aim here is to extend and apply basic ideas of graph theory and to seek for evidence of small-world-ness in beta cells from islets of Langerhans. These endocrine cells reside in the pancreatic tissue, synthesize and release insulin, and play a pivotal role in normal and pathological whole-body nutrient homeostasis [34]. Being dynamical systems, interconnected into a functional syncitium by gap junctions that connect the cytoplasm of adjacent cells, beta cells can serve as a paradigm of a biological network [35]–[39]. More importantly, the level of their interconnectedness appears to be crucial for their function. Specifically, optimal cell-to-cell electrical coupling through the gap junction protein Connexin36 (Cx36) seems to support coordinated plasma membrane depolarization, calcium signaling patterns, and insulin exocytosis in response to stimulation with secretagogues. Cell coupling has been shown to improve insulin synthesis and exocytosis, and uncoupling to lead to altered beta cell function [40]. It has been suggested that functional cell-to-cell contacts confine the stimulatory concentration range of glucose and make the otherwise heterogeneous beta cell population function in unison [41]. The idea has been put forward that this homogenization effect may explain the narrow, steep stimulus-response curve of beta cells under normal conditions, presence of hyperinsulinemia under basal glucose plasma concentrations in diabetes and in experiments where Cx36 is ablated. Finally, it may be particularly important for the prevention of hypoglycemia due to its ability to rapidly turn off insulin secretion in the face of lowering glucose [42]. Therefore, one of the central questions in beta cell physiology remains how the functional syncitium is organized under normal conditions in terms of its structure and function and how it breaks down in disease.

In the present report, we first extract beta cell functional networks by means of intracellular calcium signals from a population of beta cells, obtained by confocal laser-scanning microscopy of fluorescently labeled acute pancreas tissue slices. Next, we statistically characterize them with diagnostic tools from graph theory in view of the current understanding of complex networks. To implement the theoretical framework and measures of network theory for discernment of the organization of beta cell network, a strategy to represent physiological data in the form of graphs is required [29]. First, we measure the signal from individual network elements, providing a reliable measure of their activity. An account of the first step is provided in the chapter “Calcium Imaging”. Second, the relationship between elements is characterized and the level of similarity between their signals quantified in the chapter “Network Construction”. Third, all pairwise interactions between individual network elements are appreciated and investigated within the network-theoretical frame of reference in “Network Construction” and in “Characterization of the functional network”. Finally, the relation between known structural foundations and the obtained functional network characteristics is handled within the “Results” and “Discussion” sections, where also the importance of our findings for the understanding of the functional organization of islets of Langerhans will be discussed.

## Materials and Methods

### Ethics statement

The study was carried out in accordance with all national and European recommendations pertaining to work with experimental animals, and all efforts were made to minimize suffering of animals. The protocol was approved by the Veterinary Administration of the Republic of Slovenia (permit number: 34401-61-2009/2).

### Tissue slice preparation

Tissue slices (140 µm) were prepared from pancreata of 10–20 week old NMRI mice killed by cervical dislocation as described previously [43]. Throughout preparation and during slicing, the tissue was held in an ice-cold extracellular solution (ECS, consisting of (in mM) 125 NaCl, 26 NaHCO_3_, 6 glucose, 6 lactic acid, 3 myo-inositol, 2.5 KCl, 2 Na-pyruvate, 2 CaCl_2_, 1.25 NaH_2_PO_4_, 1 MgCl_2_, 0.5 ascorbic acid; ph 7.4) continuously bubbled with a gas mixture containing 5% CO_2_ and 95% O_2_. Slices were collected in 30 ml of HEPES-buffered saline at room temperature (HBS, consisting of (in mM) 150 NaCl, 10 HEPES, 6 glucose, 5 KCl, 2 CaCl_2_, 1 MgCl_2_; pH 7.4) before they were transferred to the dye-loading solution. All chemicals were obtained from Sigma-Aldrich (St. Louis, Missouri, USA) unless otherwise specified. 4–10 slices were simultaneously loaded with the dye in a Petri dish, exposed to ambient air, protected from light, and filled with 3.333 ml of HBS containing 6 µM Oregon Green 488 BAPTA-1 AM calcium dye (OGB-1, Invitrogen, Eugene, Oregon, USA), 0.03% Pluronic F-127 (w/v), and 0.12% dimethylsulphoxide (v/v) for 1 hour on an orbital shaker (40 turns/min) at room temperature. Uptake of OGB-1 was limited to the first two or three most superficial cell layers as described previously for pituitary slices [30], [44] and isolated islets [35], [45]. Several-fold differences in fluorescence intensity were observed between cells, most probably due to differences in viability, enzyme activity, loading, and variable extrusion of the dye. Different loading did not influence fluorescence time profiles. After staining and before measurements, the slices were kept protected from ambient light in 30 ml of fresh HBS for up to 12 hours at room temperature. HBS was exchanged every 2 hours. Individual slices were transferred to a temperature-controlled bath chamber (37°C, Luigs & Neumann, Ratingen, Germany) continuously perifused with bubbled (5% CO_2_, 95% O_2_) ECS and used in imaging experiments.

### Calcium imaging

Imaging was performed on a Leica TCS SP5 AOBS Tandem II upright confocal system using a Leica HCX APO L water immersion objective (20×, NA 1.0). OGB-1 was excited by an argon 488 nm laser and fluorescence detected by Leica HyD hybrid detector in the range of 500–650 nm (all from Leica Microsystems GmbH, Wetzlar, Germany). 8-bit 512×512 pixels images were acquired at a frequency of 0.5 Hz. To avoid recording from cells at the potentially damaged cut surface, cells at 15 µm depth or more were imaged. Optical section thickness was 4 µm which gave a reasonable trade-off between satisfactory signal strength at lowest acceptable laser power (to avoid photobleaching and prolong the maximum time of recording) and the need to keep the section thickness as thin as possible to assure recording from a single cell only. Before and after recording each time series, a higher quality fluorescence image (1024×1024 pixels) was taken and used as a reference to assess motion artefacts and regions of interest (ROIs) during analysis. Calcium kinetics was measured off-line from ROIs and exported employing Leica Application Suite Advanced Fluorescence software (Leica Microsystems GmbH, Wetzlar, Germany). Further analysis was performed using custom-made scripts in MATLAB program (The MathWorks, Inc., Massachusetts, USA). Photobleaching was accounted for by a combination of linear and exponential fit. Traces were rejected if extensive motion artefacts were observed. The fluorescence signals of OGB-1 were expressed as *F*/*F*_0_ ratios, *F*_0_ representing the initial level of fluorescence and *F* the fluorescence signal recorded at individual time points during the experiment, respectively.

### Network construction

**Figure 1:**
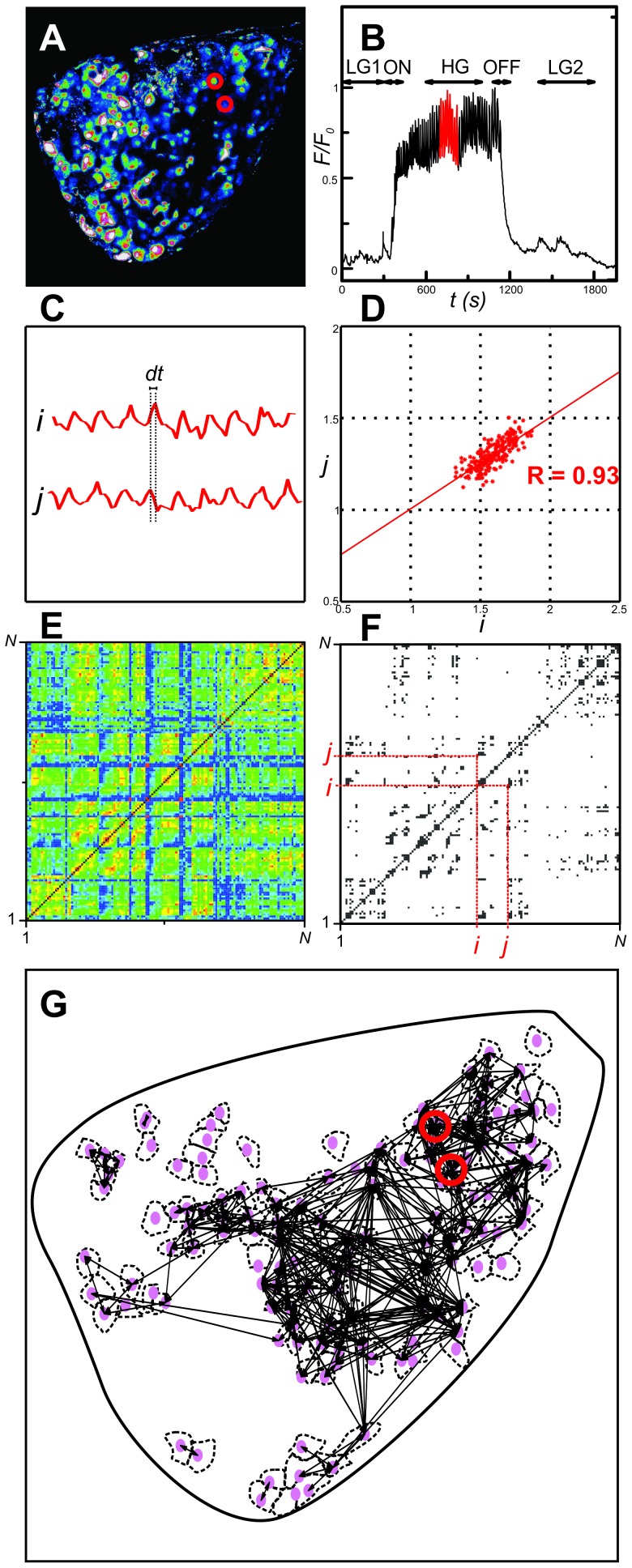
Methodology used to extract functional connectivity patterns from cytosolic Ca^2+^ traces. **A** Image of an islet of Langerhans showing the relative intensity of fluorescence signal during sustained activity (HG). Red circles indicate two cells, *i* and *j*, which we regard in continuation. **B** Temporal evolution of global calcium activity characterized by the mean-field of all beta cells in the islet. In the intervals 0≤*t*≤150 and 754≤*t*≤1960 cells were exposed to 6 mM glucose, whereas for 150≤*t*≤754 a stimulating concentration of glucose (12 mM) was applied. Arrows above the temporal trace denote five different dynamical regimes considered in this study: low glucose prior to stimulation (LG1) – 0≤*t*<300, activation of beta cells (ON) – 300≤*t*<420, sustained activity in high glucose (HG) – 600≤*t*<1000, deactivation of beta cells (OFF) – 1080≤*t*<1200, and the low glucose after stimulation (LG2) – 1400≤*t*<1800. Note that the calcium activity pattern has been normalized to the unit interval. **C** Highlighted dynamical responses of cells *i* and *j* during the HG regime. **D** Correlation diagram for fluorescence signals of the *i*-th and *j*-th cell. **E** The correlation matrix for all pair wise determined *R_ij_*. **F** The corresponding connectivity matrix (thresholded matrix, *R*_th_ = 0.75). **G** Functional connectivity map in the islet for the HG regime. Red circles indicate cells *i* and *j*.

Beta cells were distinguished from other cells on the basis of previous reports showing that cells within islets of Langerhans can reliably be identified by their type-specific calcium responses to stimulation with high glucose [46]–[48]. In cells identified as beta cells, intracellular concentration of calcium ([Ca^2+^]_i_) was low and rather stable under basal (6 mM) glucose. Upon stimulation with 12 mM glucose cells responded with a rapid increase in [Ca^2+^]_i_, followed by a sustained plateau of elevated [Ca^2+^]_i_ with superimposed oscillations (Figure 1B). In different cells these oscillations had practically identical frequencies, but were slightly out of phase (Figure 1C). After the glucose was lowered back to 6 mM, [Ca^2+^]_i_ rapidly returned to the prestimulatory level. In further analyses, five different regimes will be considered: low glucose prior to stimulation (LG1), activation of beta cells (ON), high glucose regime (HG), deactivation of beta cells (OFF), and the low glucose after stimulation (LG2) (Figure 1B). Video S1 features responses of all cells in the islet.

To obtain a graph from temporal traces of a large number of cells, we first define that two cells (ROIs) be functionally connected if the Pearson product-moment correlation between their signals over a certain time window exceeds a positive predetermined threshold value *R*_th_ [49]. In particular, we define *x_i_* as the time series to be examined, where *x_i_* stands for the fluorescence values of the *i*-th cell, rescaled to the unit interval for simplicity, even though without rescaling, identical results would be obtained. For quantification of dynamical correlations between the cells, we calculate the correlation matrix *R*, whose *ij*-th element is defined as follows:
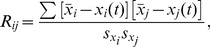
(1)Where 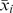 and 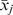 are the mean values of the time series 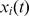 and 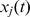, and 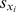 and 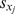 the corresponding standard deviations. If 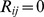 then no correlation between the *i*-th and *j*-th cell exists, whilst 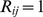 signifies completely synchronous dynamics.

The significance of obtained correlation coefficients was estimated using the t-test [50]:
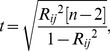
(2)where *n* signifies the number of compared data points and *R_ij_* the value of the obtained correlation coefficient. From this, the critical *R_ij_* at every sample size was calculated as follows:
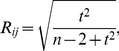
(3)All values of *R_th_* used in the following analyses are statistically significant at a level of *p*<0.001.

To convert the full connectivity matrix to a sparsely connected undirected unweighted graph, we chose a threshold *R*_th_ (above the level of significance), so that only pairs whose correlation *R_ij_* exceeds this threshold are considered to be connected [51]. By varying *R*_th_, we can adjust the mean degree of the network *k*_avg_. The methodological approach used to extract the functional connectivity patterns is schematically presented in Figure 1.

### Characterization of the functional network

In order to characterize the level of synchrony of [Ca^2+^]_i_ dynamics in the islet, we calculated the average correlation coefficient for all possible pairs of cells, *R*_avg_, which enabled us to describe the coherence of [Ca^2+^]_i_ dynamics in the islet with a single parameter. Metrics for the exploration of structural properties of the extracted networks included calculation of the clustering coefficient, the network's global efficiency, and the analysis of the degree distribution [7], [49], [50]. The clustering coefficient of the *i*-th node *C_i_* is defined as the number of existing connections between all neighbors of a node, divided by the number of all possible connections between them. The average clustering coefficient *C*_avg_ is estimated by simply averaging *C_i_* over all the vertices and thus represents a global measure for network's functional segregation [7]. The global efficiency *E*_glob_ is inversely related to the average shortest path length. The latter is defined as the average number of mediating links along the shortest path between any two nodes and reflects the traffic capacity of a network [50]. Node degree *k_i_* signifies the number of connections of the *i*-th node. Useful information about structural principles of a network can be gathered from the network's degree distribution *P*(*k*), i.e., the probability distribution of *k_i_* over the whole network. The average degree *k*_avg_ is obtained by averaging all *k_i_* over all the vertices. Especially in relatively small networks, the cumulative distribution *G*(*k*) is used for the characterization of the network, since in this manner statistical fluctuations are alleviated [50]. Notably, small-world networks are expected to simultaneously display both high integration and segregation, i.e. high global efficiency and clustering. To quantify the extent of small-world-ness in a network, one commonly compares *E*_glob_ and *C*_avg_ with the clustering coefficient *C*_rand_ and global efficiency *E*_rand_ estimated in a random network with the same number of nodes, links, and average degree as the network of interest [7], [52]. In particular, in small-world networks the ratio 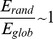 and 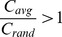. The extent of small-world-ness can be quantified with a single parameter 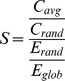, which is typically >1 [53].

## Results

**Figure 2:**
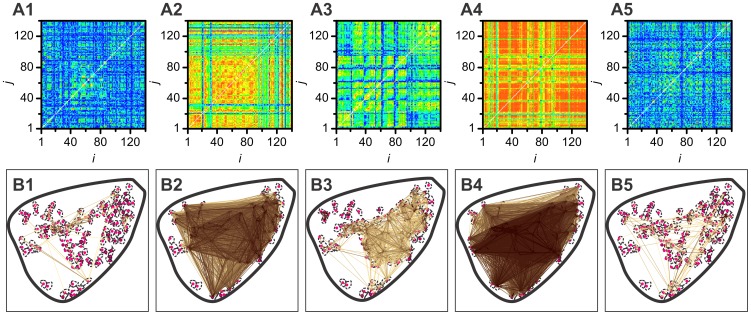
Correlation matrices *R* (upper row, images A1–A5) and network architectures (lower row, images B1–B5) for all regimes considered in this study. Upper row: correlation matrices for low glucose before stimulation (A1), activation (A2), sustained activity in high glucose (A3), deactivation (A4), and low glucose after stimulation (A5). The color mapping is linear, values of 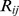 = 0.0 and 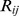 = 1.0 depicted in blue and red, respectively. Lower row: network architectures for low glucose before stimulation (B1), activation (B2), sustained activity in high glucose (B3), deactivation (B4) and low glucose after stimulation (B5). Each red dot represents the physical position of a nucleus of a glucose glucose-responsive cell inside the islet of Langerhans. Dashed lines correspond to cell outlines. Pairs of cells whose correlation exceeds *R*_th_ = 0.75 are connected with an arrow. In addition, colors of arrows signify values of *R_ij_*: linear coding between 0.75 (yellow) and 1 (dark brown).

We first calculated the linear correlation coefficient between all pairs of *N* = 140 cells from a single islet of Langerhans, whose average [Ca^2+^]_i_ response was shown in Figure 1B. The upper row in Figure 2 features the correlation matrices for all five dynamical regimes (LG1, ON, HG, OFF, LG2), as defined in the caption of Figure 1.

Evidently, the highest overall correlation between beta cells was attained during the phases of activation (ON) and deactivation (OFF). Furthermore, in the HG regime the coherence of [Ca^2+^]_i_ activity was obviously larger than in either of the low glucose regimes (LG1 and LG2) and lower than during activation and deactivation. To quantify this visual assessment, we calculated the average correlation coefficient *R*_avg_ for all five regimes. In the low stimulation regimes LG1 and LG2, the values of *R*_avg_ were 0.21 and 0.24, respectively. In the HG phase *R*_avg_ = 0.42, and in the ON and OFF phases *R*_avg_ = 0.64 and *R*_avg_ = 0.83, respectively. Next, we extracted networks from the correlation matrices as described in the Materials and Methods section. For each of the dynamical regimes we chose the same threshold *R*_th_ = 0.75. Functional networks obtained by thresholding the correlation matrix are shown in the lower row of Figure 2. As expected, the density of connections in each network was higher in regimes with well correlated cell behavior. In particular, there were more links in the HG regime than in LG1 or LG2 regimes and on average, they were darker, signifying a larger correlation under stimulation. It can also be noticed that in the network constructed from the dynamics in the low stimulation regimes, the functional connectivity was roughly independent of the Euclidean distance between the cells, whereas in the HG network there were a lot of highly correlated groups of mostly nearby cells. Furthermore, in the ON and OFF phase the resulting networks were very dense (the average degrees in the ON and OFF phases were 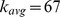 and 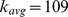, respectively), thus indicating that the cells were very synchronous in these regimes.

**Figure 3:**
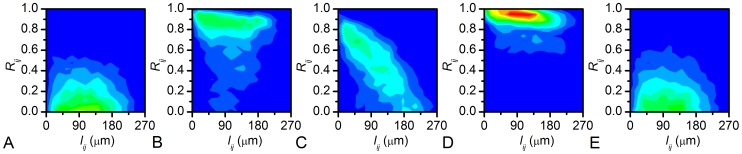
2D histograms showing the distribution of pairs of cells with regard to their Euclidean distance *l_ij_* and correlation coefficient *R_ij_*. From left to right, histograms illustrating the distribution in low glucose before stimulation (A), during activation (B), during sustained activity in high glucose (C), during deactivation (D), and in low glucose after stimulation (E) are presented. The color profile is linear and the same in all panels. Blue depicts 0 and red 520 pairs of connected cells in a given interval.

To find out whether network analysis can provide evidence for [Ca^2+^]_i_ waves as the mechanistic substrate of beta cell synchronization, we examined the relationship between the Euclidean distances *l_ij_* between beta cells and their dynamical correlations in more detail, by making use of 2D binning. More specifically, we calculated the number of cell pairs which fell within a given range of *l_ij_* and *R_ij_*. 2D histograms shown in Figure 3 reveal that in the HG regime there is a strong tendency of nearby cells to be much better correlated with each other than with the remote ones. Interestingly, in none of the other regimes a similarly convincing trend could be noticed.

**Figure 4:**
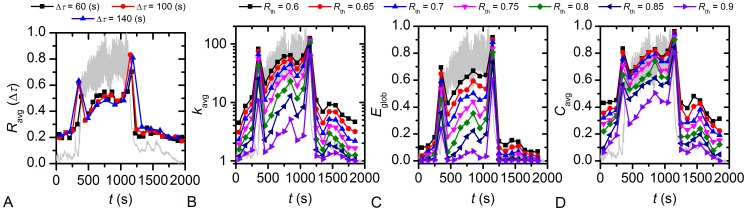
Temporal evolution of the average correlation coefficient, average degree, global network efficiency, and average clustering coefficient under stimulation with glucose. In this figure, temporal changes of the average correlation coefficient (A), average degree (B), the global network efficiency (C) and the average clustering coefficient (D) are presented on a background of the mean-field calcium signal. Parameters calculated for network architectures obtained at different thresholds *R*_th_ are color-coded as indicated in the figures. In panels B–D Δ*τ* = 100 s. For this Δ*τ*, the number of degrees of freedom is such that significance at a level of p<0.001 is achieved for all *R_ij_* exceeding 0.62. Values of all of the parameters increase during activation and even more so under deactivation. During sustained activity, values are higher than before or after exposure to glucose. During activity, values tend to increase until the solution containing nonstimulatory glucose reaches the cells.

Next we explored how the characteristics of the functional network evolved with time. A visualization of the rewiring process of functional connections within an islet throughout different phases can be found in Video S2. Furthermore, we calculated the evolution of *R*_avg_ as well as of several measures for network characterization – the average degree *k*_avg_, the global efficiency *E*_glob_, and the average clustering coefficient *C*_avg_, in order to trace the evolution of the functional network structure. For this purpose, we divided the time series of individual cells into intervals of length Δ*τ* and calculated the quantities on given intervals, i.e. sliding windows. Results presented in Figure 4 reveal that *R*_avg_, *k*_avg_, *E*_glob_, and *C*_avg_ all displayed a similar behaviour, with a peak during activation and deactivation, and with values during activity being higher than during non-stimulatory conditions. The choice of duration of Δ*τ* does not have a significant impact on the calculated *R*_avg_ (Figure 4A). Furthermore, it can be noticed that the network measures are qualitatively independent of the chosen threshold *R*_th_, since with increasing *R*_th_ only a monotonous decrease of all quantities of interest is observed. To get a more precise insight into the temporal evolution of correlation between cell pairs we additionally calculated the sliding correlation with overlapping intervals. In particular, we calculated the average correlation within a constant interval Δ*τ* = 100 s, whereby the interval was being slided along time series with a step Δ*τ*′ = 20 s. Results showing a more detailed evolution of the average correlation between beta cells are presented in Figure S1.

In order to strengthen the reliability of our findings, the calculations that follow rely on 9 functional networks having alltogether 562 nodes, that were constructed in 9 islets of Langerhans (from 6 animals). In all slices comparable [Ca^2+^]_i_ activity patterns were detected (the protocols of stimulation with glucose were as similar as practically achievable), so that the same five regimes could be identified in all of them.

First, we verified if the abovementioned findings can be generalized to other islets. For that purpose we calculated the distribution of pairs of cells that fall within a given range of *R_ij_* in all five regimes. Results in Figure S2 clearly support the genaralization of our results, since also in other islets the same trend is observed. The ON and OFF phases exhibited very high correlations and the correlations observed in the HG regime were higher compared with the LG1 and LG2 regimes.

Extremely high correlations in the ON and OFF phases which were found in signals from all slices led to very densely connected networks (see lower row in Figure 2) whose structural properties are quite similar to those in a fully connected graph. This, we ascribe to the fact that during the ON and OFF phases, every beta cell is independently driven by the external stimulus, i.e. by the elevation or lowering of the concentration of glucose (see Discussion). Due to this and because we were particularly interested in the network behavior during activity (i.e. during the HG regime), in the following analyses we focused on the analysis of networks extracted from the LG1, HG, and LG2 regimes. The calculation of *R*_avg_ between all pairs of cells within a given interval of physical distance confirmed the previously described dependence of correlation on Euclidean distance during the HG regime (Figure S3).

**Figure 5:**
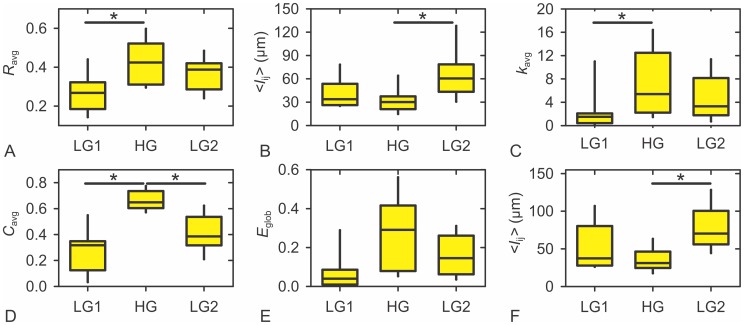
Characteristics of functional networks of beta cells in 9 islets of Langerhans for three of the five regimes analyzed. **A** The average correlation coefficient. **B** The average physical distance between connected cells. **C** The average node degree. **D** The average clustering coefficient. **E** The global efficiency. **F** the average lengths of connections originating from 20% of the cells with the highest degree. In all panels red circles denote the median values, whereas the yellow floating columns signify the broadness of intervals within which the values were detected in all 9 islets. Significant differences obtained after pairwise *post hoc* Wilcoxon signed-rank tests are indicated by asterisks.

To describe the average properties of the functional networks (9 datasets) in more detail, we calculated for the LG1, HG, and LG2 regimes the respective average correlation coefficient *R*_avg_ (as a reference), the average physical distance between connected cells *l_ij_*, the average degree *k*_avg_, the average clustering coefficient *C*_avg_, the global efficiency *E*_glob_, and the average lengths of connections that originate from 20% of the most connected cells in the slice. Results are presented in Figure 5. Comparisons between regimes were done performing Friedman's ANOVA in the first step and *post hoc* Wilcoxon signed-rank test in the second to compare LG1 with HG, HG with LG2, and LG1 with LG2. A Bonferroni correction for the number of *post hoc* pairwise comparisons was applied and so all differences are reported at the level of significance p<0.05/3 = 0.0167. For all 6 parameters the differences between groups were significant. Significant differences obtained after pairwise *post hoc* comparisons are indicated by asterisks. During HG regime, *R*_avg_ was significantly higher than in LG1, *l_ij_* significantly shorter than in LG2, *k*_avg_ significantly higher than in LG1, *C*_avg_ significantly higher than in either of the LG regimes, further supporting the results obtained so far. To check whether the nodes with the highest degrees connect predominantly to physically close or distant cells, we calculated the average lengths of connections originating from 20% of the cells with the highest degree. The latter were significantly shorter in HG than in LG2. Additionally, by comparing panels B and F it appears that the most connected nodes do not display any preference for either short- or long-range connections in neither of the regimes considered. For *E*_glob_, differences between HG and LG1 and HG and LG2 were not statistically significant (*p*<0.021 for LG1 vs. HG and *p*<0.086 for HG vs. LG2), but a trend towards higher efficiency in HG is clearly visible. Notably, the differences between both regimes in low glucose, LG1 and LG2, were never statistically significant, strongly supporting the view that the effect of stimulation was largely reversible.

**Figure 6:**
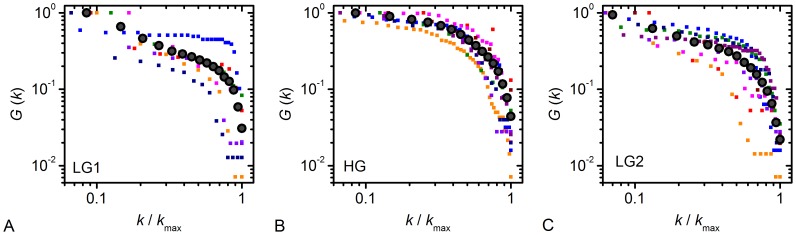
Cumulative degree distributions of functional networks for three of the five regimes analyzed. Values obtained from all 9 islets are plotted in different colors and the larger black circles indicate their average for low glucose prior to stimulation (A), high glucose (B), and low glucose after stimulation (C). The node degree distributions decay roughly linearly before a drop-off in the tail, thereby indicating a broad-scale nature of the networks.

To determine the scale of our networks we plotted the cumulative degree distribution (Figure 6). In order to be able to combine data from 9 islets with different numbers of responsive cells, we normalized *k* of each cell to the maximal node degree *k*_max_ in the respective islet. In this manner we obliterated the effect of different network sizes and resulting maximal degrees and were thus able to focus on the shape of the distribution only. We drew the cumulative distributions for all 9 islets and three of the five regimes (LG1, HG and LG2), which were then averaged (dark circles). It can be observed that in all three regimes the averaged values decay roughly linearly in the double logarithmic plot before a drop-off in the tail. These results indicate that the functional connectivity between beta cells displays a broad-scale nature. Namely, such connectivity distributions are characterized by a power-law regime followed by a sharp cutoff [9]. To quantify this visual evaluation, we fitted the data with three possible models: a power-law, an exponential decay and an exponentially decaying power-law. Goodness-of-fit was tested using the coefficient of determination *r* ^2^, whereby a better fit is indicated by a value closer to 1. Of all three model functions, the exponentially truncated power law was the best-fitting model for the cumulative degree distributions in all three regimes. It gave *r* ^2^ values of 0.99, 0.99 and 0.98 for the LG1, HG and LG2 regime, respectively. The other two fitting options yielded *r* ^2^ values ranging from 0.8 to 0.98, thus validating that the observed degree distributions are best described by truncated power-laws which is a characteristic of broad-scale networks.

**Figure 7:**
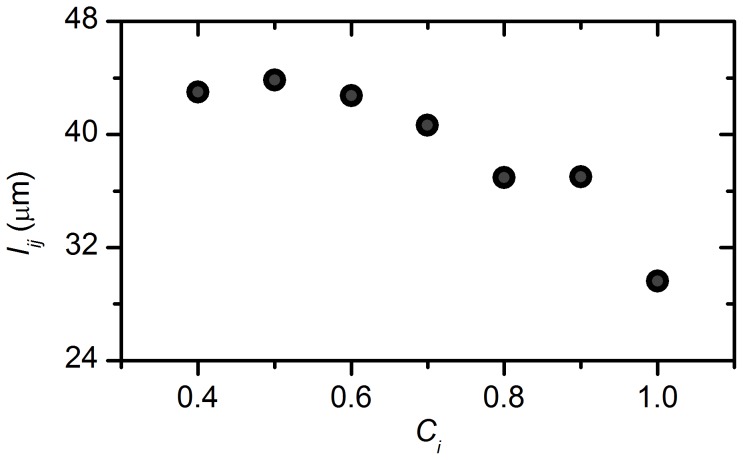
Average length of links as a function of the local clustering coefficient. Plotted are the values obtained for the HG regime.

Since in the HG regime a clear increase in correlation between the nearest cells was visible, we further checked whether clustering in this regime was also related to the physical distance between cells. For this purpose we calculated the average length of connections <*l_ij_*> originating from a cell with a given local clustering coefficient *C_i_*. The obtained values were then averaged over all cells whose local clustering fell in a given range of *C_i_*. In Figure 7, we see that in the HG regime, high clustering was indeed supported by short links. Taking into account a typical beta cell diameter of 20 µm, these links existed between direct neighbors or cells separated by the characteristic distance of one cell at most. As detailed in Discussion, this distance was not necessarily spanned by a beta cell. To sum up, the clustering was negatively related with the connection distance, meaning that the cells with long connections were linked with remote regions that were otherwise not connected to each other.

Finally, to diagnose small-world properties in the networks we compared *C*_avg_ and *E*_glob_ with the same metrics estimated in a random graph configured with the same number of nodes and mean degree *k*_rand_, as the network of interest [7], [52]. If the ratios 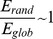 and 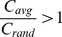, a network exhibits a large extent of small-world-ness (see Materials and Methods). Table 1 summarizes the results for the networks in all three regimes of interest (LG1, HG, LG2). Note that the median values for all 9 networks as well as of the values of the corresponding random networks are shown. The results indicate that the functional connectivity patterns between beta cells possess properties of small-world networks. These features are especially well pronounced in networks extracted from cellular responses in the HG regime. Under stimulating conditions, the demand of having efficient communication abilities seems to be particularly conspicuous. The increase in the extent of small-world-ness *S* that we observed upon stimulation with glucose occurred predominantly due to an increase in global efficiency, which reflects shortening of the average path length.

**Table 1:** Table of small-world-ness values.

	*C*_avg_	*E*_glob_	*C*_rand_	*E*_rand_	*C*_avg_/C_rand_	*E*_rand_/E_glob_	*S*
LG1	0.32	0.07	0.06	0.21	5.29	2.84	1.86
HG	0.67	0.29	0.12	0.41	5.59	1.41	3.96
LG2	0.39	0.15	0.09	0.36	4.28	2.48	1.73

Median values of statistical parameters indicative of small-world properties in 9 islets from 6 animals, for the functional networks obtained for low glucose before stimulation (LG1), high glucose (HG), and low glucose after stimulation (LG2). *C*_avg_ - average clustering coefficient for the real network considered in this study, *E*_glob_ - global efficiency for the real network considered in this study, *C*_rand_ - average clustering for the reference random network, *E*_rand_ - global efficiency for the reference random network, *C*_avg_/*C*_rand_ - ratio between the clustering coefficients of the real world and reference random network, *E*_rand_/*E*_glob_ - ratio between the global efficiencies of the reference random and the real world network, S- ratio between the latter two ratios. Note that in HG, *S* is higher than in LG1 and LG2 predominantly due to a higher *E*_glob_.

## Discussion

A number of previous studies employing tools developed in the frame of complex network theory have suggested a strong global structure-function correlation and the importance of small-world features for optimal functional connectivity [7], [53]–[55]. Additionally, the level of synchronization of Ca^2+^ signals between different regions of isolated mouse and human islets has been assessed [56], [57]. However, gaining insight into the beta cell network organization at the level of a large number of individual cells has so far been prevented by the inability of the classical physiological model of isolated islets of Langerhans combined with calcium imaging techniques to access cells in the core of an islet, where, at least in mice, the majority of beta cells are located, due to uneven loading of calcium indicators, presence of several cell layers, and partially also by the lower spatial resolution of CCD-camera based recording setups [35], [46], [56], [58], [59]. In the present work, we set out to extract functional networks of beta cells from experimental data obtained on a large number of individual cells from all cell layers of an islet. By employing our experimental preparation, to the best of our knowledge, we showed for the first time that laser-scanning confocal microscopy of fluorescently labeled beta cells from acute pancreatic tissue slices gives data of sufficient temporal and spatial resolution to make feasible the analysis of cells from a whole cross-sectional surface. The functional networks obtained from correlated time series of beta cells under basal and stimulated conditions support the view that the activity of different beta cells is not completely synchronous but synchronized to a level determined by [Ca^2+^]_i_ waves spreading across the syncitium of beta cells. From our study, the latter seems to obey small-world organizing principles. Our work also opened some methodological questions that will be discussed here and will need to be addressed during future efforts in this field.

### Calcium dynamics

Qualitatively, the dynamics of calcium responses we obtained were in good agreement with what has been described in isolated islets [56], [60], [61]. Since this is the first report of confocal calcium imaging in the tissue slice preparation, which in its own right constitutes a novel experimental approach to study beta cell physiology under conditions very closely resembling the *in vivo* environment, a more extensive and quantitative description of beta cell behavior under our experimental setting will be provided elsewhere.

In short, previous studies employing calcium imaging with subsequent identification of types of cells by immunocytochemistry have shown that cells can reliably be identified by their type-specific calcium responses to stimulation with high glucose [46]–[48]. Taking advantage of this finding, we included in our analyses only cells that showed responses characteristic of beta cells, i.e. cells that showed no oscillations in low glucose, responded to stimulation with high glucose with oscillations superimposed on a sustained rise in [Ca^2+^]_i_, and displayed a return of [Ca^2+^]_i_ back to prestimulatory levels after the end of stimulation. Sometimes, transient increases of [Ca^2+^]_i_ could be observed in cells functionally identified as beta cells after [Ca^2+^]_i_ had reached basal levels (Figure 1B). Cells displaying oscillations in low glucose before stimulation (characteristic of alpha and delta cells) could be detected at the periphery of islets. This is in agreement with previous studies showing sequestration of non beta cells to the outermost layers in mice [62], [63]. All of the latter cells were excluded from our analyses.

### Functional connectivity

Upon stimulation with glucose, the cytosolic calcium increased and after lowering the glucose to basal concentration, it rapidly returned to the prestimulatory level. Both activation and deactivation were manifested as a prominent rise of the average correlation between cell responses, with more cells reaching the threshold value of *R*_th_, leading to a change in network structure characterized by more connections. The density of connections in graphs obtained in ON and OFF regimes reflects that practically all of the cells considered in analysis responded to the stimulus and that this response was very well synchronized. Deactivation, however, was even more synchronized than activation and therefore, the OFF graph displayed an even higher correlation. Time lags between individual cells during activation and deactivation were at most a few tens of seconds. Moreover, despite large changes in [Ca^2+^]_i_ during these two phases, cells that were close to the first-responding cells did not respond with an increase in [Ca^2+^]_i_ shortly after. In line with this, no [Ca^2+^]_i_ waves could be observed during the ON and OFF phases, but cells responding individually or in small groups, with no clear dependence on the Euclidean distance from the first responders. We therefore conclude that during both activation and deactivation cells responded in synchrony not because of a large degree of gap junctional coupling during these two transient phases, but because each cell individually was forced to activate by high glucose during beginning and to deactivate by lowering glucose at the end of stimulation. The large amplitudes and long durations, in combination with the chosen length of sampling interval, led to a high correlation during both transitory phases, despite time lags between different cells.

The larger correlation during deactivation in comparison with activation is due to shorter time delays observed during this phase. In an electrically coupled syncitium, the efficiency of spread of electrical signals is expected to be determined by the gap junctional and membrane conductance, such that the higher the gap junctional conductance and the lower the membrane conductance, the more efficient the transmission of stimuli. Therefore, it is not surprising that deactivation, starting from a well coupled state, was more synchronous than activation that started from a state where membrane conductance of cells that were not yet metabolically activated was high due to open K_ATP_ channels. The high correlation during deactivation supports the experimentally observed rapid termination of insulin secretion in face of lowering glucose which probably helps prevent hypoglycemic episodes. The level of synchronization seemed independent of Euclidean distance for both activation and deactivation. This is probably due to poor coupling during activation with cells in different parts of an islet responding independently from each other, and due to deactivation starting simultaneously in more than one part of the islet.

In contrast, during HG regime when cells displayed sustained regular oscillations, the level of synchronization strongly depended on physical distance, with correlation almost linearly falling with increasing distance between cells. Obviously, this finding practically excludes the possibility that beta cells in different parts of an islet show completely synchronous activity and strongly supports the idea that calcium waves spread across the islet with a speed sufficient to allow for the presence of oscillations in the mean field signal, occurring with practically the same frequency as in individual cells [64]–[66]. Interestingly, at every *R_ij_*, the range of distances represented in Figure 3C is rather broad, pointing to the possibility that some cells are very well synchronized despite large internodal distances, implying, in turn, that the velocities of the calcium waves probably encompass a broad range of values. From this, it follows that not all beta cells are equal in terms of conducting calcium waves and that a minority of faster than average (or majority) conducting cells could ensure a rapid activation of the whole islet. This prediction finds important support after looking at the obtained efficiency, which together with the relatively high clustering coefficient in comparison with the random graph shows that the beta cell syncitium functions as a small world. During the first half of the plateau phase, *R*_avg_, *k*_avg_, *C*_avg_, and *E*_glob_ all tend to increase with time, highlighting that the synchronization gradually improves, possibly due to an increase in gap junctional conductance or due to a relative increase in gap junctional conductance in comparison with the membrane conductance which is expected to decrease in beta cells with rising glucose and ATP (Figure 4). The same reasoning could also serve to explain the decrease in the above parameters observed after approximately 900 seconds, which coincides well with the time when glucose and intracellular ATP concentration are supposed to start decreasing. The observed small-world properties might prove crucial for a rapid activation of beta cells after exposure to higher glucose and for their synchronized activity during stimulation. Additionally, the high clustering could confer resilience to dysfunction of individual cells. From the relationship between physical distance and clustering and taking into account that a typical beta cell is around 20 µm in diameter it becomes clear that the highest clustering occurs in cliques of closest cells.

In contrast to neurons that possess cellular processes encompassing a whole range of possible lengths and enable these cells physical contact with nearest neighbors as well as with cells in more distant parts of an organ or even body, beta cells are typically of a polyhedral shape and make direct physical contact with their nearest neighbors only. From a graph-theoretical perspective, at first glance an islet of Langerhans might seem to be an example of a regular network where all cells are only coupled to their nearest neighbors [7], [67]. This view is even integrated in most of the modeling work on islets of Langerhans [68]. Therefore, whilst for neuronal populations and organ systems made of neurons, small-world-ness seems rather implicit, it can only hardly be predicted for beta cells embedded in an islet of Langerhans. However, bearing in mind that structural and functional networks are fundamentally different and considering more closely the way beta cells are coupled functionally might provide an explanation for the observed, unexpectedly short path length. It is widely recognized that beta cell electrical activity and calcium oscillations that supposedly underlay the pulsatile insulin secretion are synchronized via gap junctions [35]–[38], [69]–[74]. Here, we propose that a significantly higher conductivity than average in a relatively small proportion of beta cells could defy strict physical boundaries of direct contact with nearest neighbors only and make these cells serve as hubs, facilitating the signal spread between distant parts of an islet. Some studies to date on dispersed pairs of cells [70], microdissected islets [75], and isolated islets [35] partly support evidence in favor of this possibility. In addition, it has been demonstrated that spatial inhomogeneities, such as variations of glucose concentration across the islet, can coordinate or suppress the propagation of waves [76] and that heterogeneity of the coupling strength can modulate excitation waves spreading throughout islets of Langerhans [77]. However, a larger number of randomly chosen beta cells in tissue slices will have to be investigated to explore this idea into further detail. Noteworthy, in case that there is a rather constant specific conductivity (per membrane area), the heterogeneity of cell surface sizes should be explored further to seek for evidence for a possibly broad distribution in this regard.

Another possible explanation for the small-world-ness of a structurally regular network is that our preparation spares some parasympathetic ganglia and postganglionic nerve fibers and that the synchronization of distant parts is mediated via nerve endings. Also, the possibility of a paracrine, fast-acting synchronizing mediator is worth exploring further.

Finally, we wish to emphasize that the presence of functional connections between remote cells does not imply the existence of direct physical connections between them but merely a high degree of similarity between their [Ca^2+^]_i_ signals. The latter can be the consequence of a high degree of gap junctional coupling between all the cells interposed between the observed pair of cells, the presence of neuronal connections between cells or some other as yet unidentified mechanism.

### Methodological issues

Some technical constraints encountered during this study merit additional attention, since they provide a fertile ground for future improvements, and more importantly, because they influence the interpretation of results. During staining the dye molecules reached the cells via diffusion, consequently the outermost cell layer was stained best. Unfortunately, this is also the layer that was separated from the rest of the islet by the razor blade and therefore experienced most of mechanical trauma during the cutting step. An acceptable trade-off between maximum dye uptake and minimum mechanical damage was achieved by setting the focal plane so as to record from the second or third layer of cells below the outermost one. Here, not all of the cells covering the cross-sectional surface had taken up the calcium dye and second, of those that showed stable basal calcium signals, not all responded to elevated glucose. This could be attributed to suboptimal dye concentration or time of incubation. Also, even in this somewhat deeper layer, some residual damage due to mechanical trauma, hypoxia, temperature susceptibility, toxicity of the detergent or the dye itself etc., cannot be excluded.

An islet of Langerhans is made up of at least five different types of endocrine cells (alpha, beta, delta, epsilon, PP) and non-endocrine cells that include nerves, dendritic cells, macrophages, fibroblasts, vascular endothelial cells, and pericytes [78]. Of the endocrine cells, beta cells are by and large the most predominant type of cells and are practically the only type of cell present in the central portion of islets in mice [62]. Since our analyses relied on responsive beta cells, it is not surprising that most of the cells in Figure 1 are located in the center of the islet. Here, beta cells by no means constituted a confluent surface but formed groups of a few cells that were separated from each other by areas that did not show responses characteristic of beta cells. We believe that aside from poorly loaded and damaged beta cells, these areas correspond to capillaries. Namely, islets of Langerhans are highly vascularized organs, with each beta cell being in direct contact with a capillary [79]. Apart from capillaries, towards the periphery arterioles, lymphatic vessels, and other endocrine cells contribute to the observed rarefaction of beta cells. However, between any two cells on a plane, there are a great number of possible paths from one to the other that go via cells lying in deeper layers from which no direct recordings could be made. Therefore, at first glance, it may seem hardly conceivable that a link connects two physically distant but not two physically more proximate cells, but after taking into account the above considerations, this cannot only be explained but seems rather predictable. Non-excitable and non-conducting cells can influence the patterns of excitation. Thus, in addition to the already mentioned heterogeneity of beta cells, they might contribute to the observed network properties.

Moreover, due to the rather homogeneous and radially symmetrical islet architecture regarding distribution of endocrine cells as well as capillaries, we believe that the findings obtained from a certain transversal section do not importantly differ from the ones we would obtain in a given islet in any other possible transversal section of a comparable diameter. Due to this and due to the consistency of findings from different islets, we strongly doubt that the presented results were significantly influenced by the technical limitations of our study.

Lastly, it needs to be acknowledged that we studied a two-dimensional cross-section of a three-dimensional organ, which implies that our findings may not firmly reflect the functional connectivity between beta cells in the whole Islet. Unfortunately, with the existing technology whole-islet calcium imaging is hardly feasible. However, if we take into account that we scrutinize a system with a rather isotropic cytoarchitecture and that in complex networks with long-range connections (such as small-world networks) the dimension of the space is more or less irrelevant [80], we assume that qualitatively similar results would be obtained, even if we were able to simultaneously trace the dynamics of all cells in the whole Islet. Shall whole-islet calcium imaging be feasible one day, most probably by employing two-photon excitation of protein Ca^2+^ indicators, selectively expressed in beta cells of transgenic animals, it would become possible to experimentally verify these predictions.

### Conclusion

Employing a novel approach to *in situ* study of islet physiology we presented evidence that the functional connectivity of beta cells reconstructed from cytosolic calcium time traces displays characteristics of a small-world network, which might reflect an optimal organization in terms of rapid synchronization, local redundance of activating signals, resilience to certain types of damage, as well as a balance between local and global processing. We hope that our work will prompt researchers to seek for a structural explanation of the observed small-world-ness of islets of Langerhans, as well as to use the approach presented in this study also on other tissues to find out whether this type of organization represents a principle, ubiquitous in assemblies of coupled cells.

## Supporting Information

Figure S1 **Temporal evolution of the average correlation coefficient** ***R′*** **_avg_ with overlapping intervals.** Constant time interval Δ*τ* = 100 s was being slided along time series with a step Δ*τ′* = 20 s.
(TIF)

Figure S2 **Distribution of correlation coefficients for 562 cells from 9 different slices.** Distribution of pairs of cells that fall within a given range of *R_ij_* were calculated for the five different regimes considered in this study: low glucose before stimulation (LG1), activation (ON), high glucose (HG), deactivation (OFF), and low glucose after stimulation (LG2), color-coded as indicated in the figure. Evidently, in the ON and OFF phases most of the pairs of cells exhibited a very high correlation (44% pairs in the ON phase and 64% in the OFF phase have *R_ij_*>0.8). Furthermore, a clear shift to higher correlations was observed in the HG regime in comparison to LG1 and LG2 regimes, thus indicating that cells were more synchronized with each other during stimulatory than during unstimulatory conditions.
(TIF)

Figure S3 **The average correlation coefficient** ***R*** **_avg_ for all pairs of the 562 cells from 9 different slices within a given interval of Euclidean distance.** Three of the five regimes are considered: LG1, HG and LG2. Evidently, in the low stimulation regimes the coherence of cellular activity was weakly dependent on the physical distance, whereas on the other hand, in the HG regime an obvious trend of nearby cells being much better correlated than distant ones is observed.
(TIF)

Video S1 **Cytosolic calcium in cells of an islet of Langerhans before, during, and after exposure to 12 mM glucose.** LG1, ON, HG, OFF, and LG2 indicate the five different dynamical regimes considered in this study: low glucose prior to stimulation (LG1) – 0≤*t*<300, activation of beta-cells (ON) – 300≤*t*<420, sustained activity in high glucose (HG) – 600≤*t*<1000, deactivation of beta-cells (OFF) – 1080≤*t*<1200, and the low glucose after stimulation (LG2) – 1400≤*t*<1800. The red dot indicates the interval during which the cells were exposed to 12 mM glucose. During the time intervals before and after exposure to 12 mM glucose, cells were perifused with a solution containing 6 mM glucose.
(WMV)

Video S2 **Functional networks and the corresponding 2D histograms obtained before, during, and after exposure to 12 mM glucose.** In this movie, temporal changes of the architectures of functional networks and of 2D histograms can be seen in steps of Δ*τ* = 100 seconds. Note how the density and color of connections change throughout the experiment. During the ON and OFF phases, the respective graphs are almost fully connected. In 2D histograms, there is a strong tendency of nearby cells to be much better correlated with each other than with the remote ones during the HG regime. Interestingly, in none of the other regimes a similar trend can be noticed, as no relationship exists between the distribution of correlation coefficients and the Euclidean distance.
(WMV)
