# Supplementary figures and images for: Functional Connectivity in Islets of Langerhans from Mouse Pancreas Tissue Slices
Source: PLoS Comput Biol. 2013 Feb 28;9(2):e1002923. doi: 10.1371/journal.pcbi.1002923 (PMC3585390; doi:10.1371/journal.pcbi.1002923)

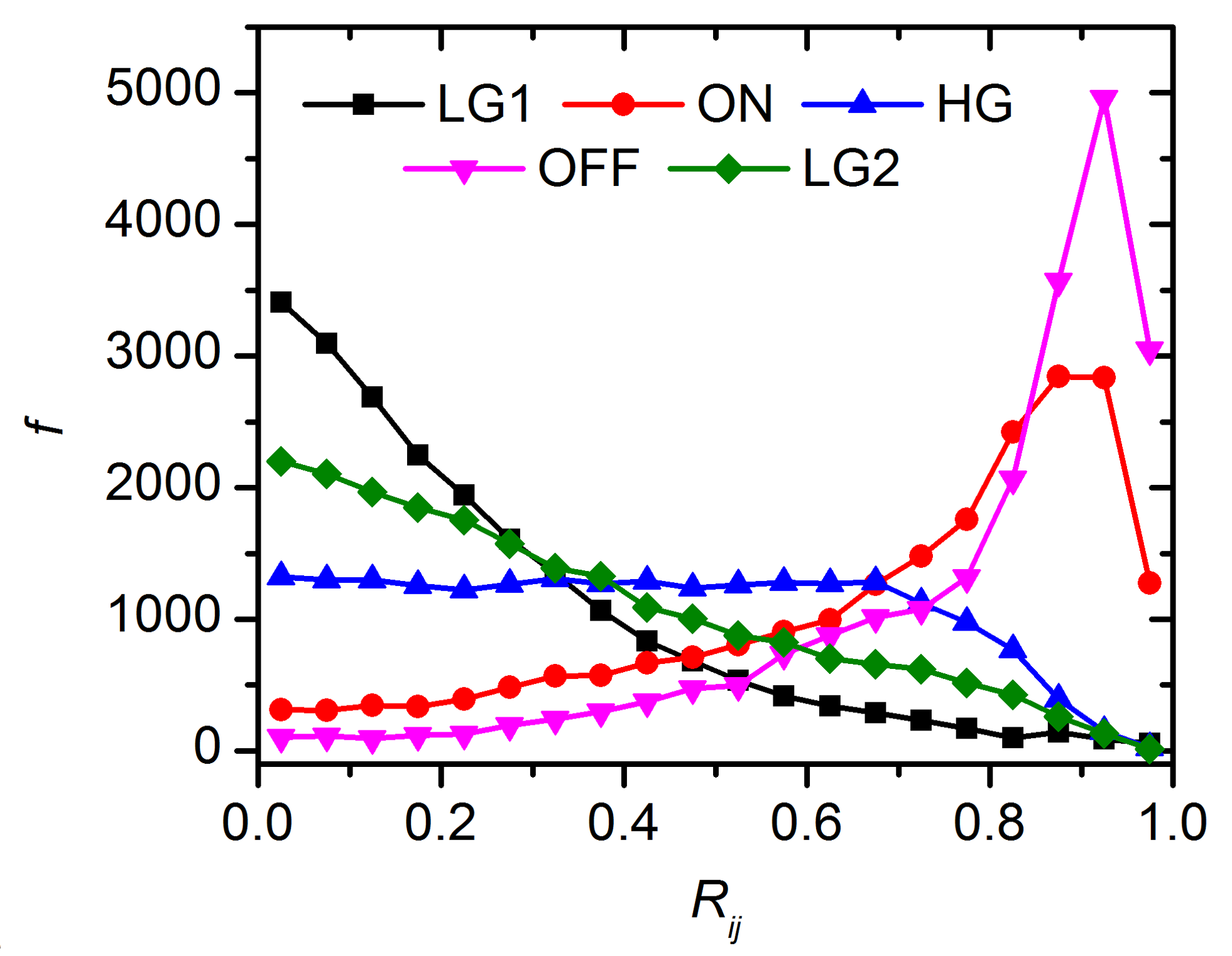

Supplement: Figure S2 — Distribution of correlation coefficients for 562 cells from 9 different slices. Distribution of pairs of cells that fall within a given range of Rij were calculated for the five different regimes considered in this study: low glucose before stimulation (LG1), activation (ON), high glucose (HG), deactivation (OFF), and low glucose after stimulation (LG2), color-coded as indicated in the figure. Evidently, in the ON and OFF phases most of the pairs of cells exhibited a very high correlation (44% pairs in the ON phase and 64% in the OFF phase have Rij>0.8). Furthermore, a clear shift to higher correlations was observed in the HG regime in comparison to LG1 and LG2 regimes, thus indicating that cells were more synchronized with each other during stimulatory than during unstimulatory conditions. (TIF) [file pcbi.1002923.s002.tif]
